# Determination and Pharmacokinetic Study of Gentiopicroside, Geniposide, Baicalin, and Swertiamarin in Chinese Herbal Formulae after Oral Administration in Rats by LC-MS/MS

**DOI:** 10.3390/molecules191221560

**Published:** 2014-12-22

**Authors:** Chia-Ming Lu, Lie-Chwen Lin, Tung-Hu Tsai

**Affiliations:** 1 Institute of Traditional Medicine, School of Medicine, National Yang-Ming University, No. 155, Sec. 2, Li-Nong St, Beitou District, Taipei 11221, Taiwan; E-Mails: a121060@gmail.com (C.-M.L.); lclin@nricm.edu.tw (L.-C.L.); 2 National Research Institute of Chinese Medicine, No. 155-1, Sec. 2, Li-Nong St., Beitou District, Taipei 11221, Taiwan; 3 Graduate Institute of Acupuncture Science, China Medical University, No. 91, Hsueh-Shih Road, Taichung 404, Taiwan; 4 School of Pharmacy, College of Pharmacy, Kaohsiung Medical University, No. 100, Shih-Chuan 1st Road, Kaohsiung 80708, Taiwan; 5 Department of Education and Research, Taipei City Hospital, No.145, Zhengzhou Rd., Datong Dist., Taipei 103, Taiwan

**Keywords:** phytochemical analysis, LC-MS/MS, herbal medicine, pharmacokinetics, traditional Chinese medicine

## Abstract

A sensitive and efficient liquid chromatography-tandem mass spectrometry (LC-MS/MS) method was developed for the simultaneous determination of gentiopicroside, geniposide, baicalin, and swertiamarin in rat plasma. To avoid the stress caused by restraint or anesthesia, a freely moving rat model was used to investigate the pharmacokinetics of herbal medicine after the administration of a traditional Chinese herbal prescription of Long-Dan-Xie-Gan-Tang (10 g/kg, p.o.). Analytes were separated by a C18 column with a gradient system of methanol–water containing 1 mM ammonium acetate with 0.1% formic acid. The linear ranges were 10–500 ng/mL for gentiopicroside, geniposide, and baicalin, and 5–250 ng/mL for swertiamarin in biological samples. The intra- and inter-day precision (relative standard deviation) ranged from 0.9% to 11.4% and 0.3% to 14.4%, respectively. The accuracy (relative error) was from −6.3% to 10.1% at all quality control levels. The analytical system provided adequate matrix effect and recovery with good precision and accuracy. The pharmacokinetic data demonstrated that the area under concentration-time curve (AUC) values of gentiopicroside, geniposide, baicalin, and swertiamarin were 1417 ± 83.8, 302 ± 25.8, 753 ± 86.2, and 2.5 ± 0.1 min µg/mL. The pharmacokinetic profiles provide constructive information for the dosage regimen of herbal medicine and also contribute to elucidate the absorption mechanism in herbal applications and pharmacological experiments.

## 1. Introduction

Long-Dan-Xie-Gan-Tang (LDXGT) is one of the best known traditional Chinese herbal prescriptions for the treatment of chronic hepatitis, jaundice, cystitis, and conjunctival congestion earache, as well as scrotum and extremitas inferior eczema [1,2]. According to the guidelines on Chinese herbal prescriptions (2013) from the Department of Chinese Medicine and Pharmacy, LDXGT consists of the following 10 herbal medicines: roots of *Gentiana scabra* (Chinese herbal name: long-dan-cao), roots of *Scutellaria baicalensis* (Chinese herbal name: huang-qin), fruits of *Gardenia jasminoides* (Chinese herbal name: zhi-zi), tubers of *Alisma orientalis* (Chinese herbal name: ze-xie), stems of *Clematis montana* (Chinese herbal name: mu-tong), seeds of *Plantago asiatica* (Chinese herbal name: che-qian-zi), roots of *Angelica sinensis* (Chinese herbal name: dang-gui), roots of *Rhemannia glutinosa* (Chinese herbal name: di-huang), roots of *Bupleurum chinense* (Chinese herbal name: chai-hu), and roots or rhizomes of *Glycyrrhiza uralensis* (Chinese herbal name: gan-cao), with a weight ratio of 4:2:2:4:2:2:2:2:4:2.

**Figure 1:**
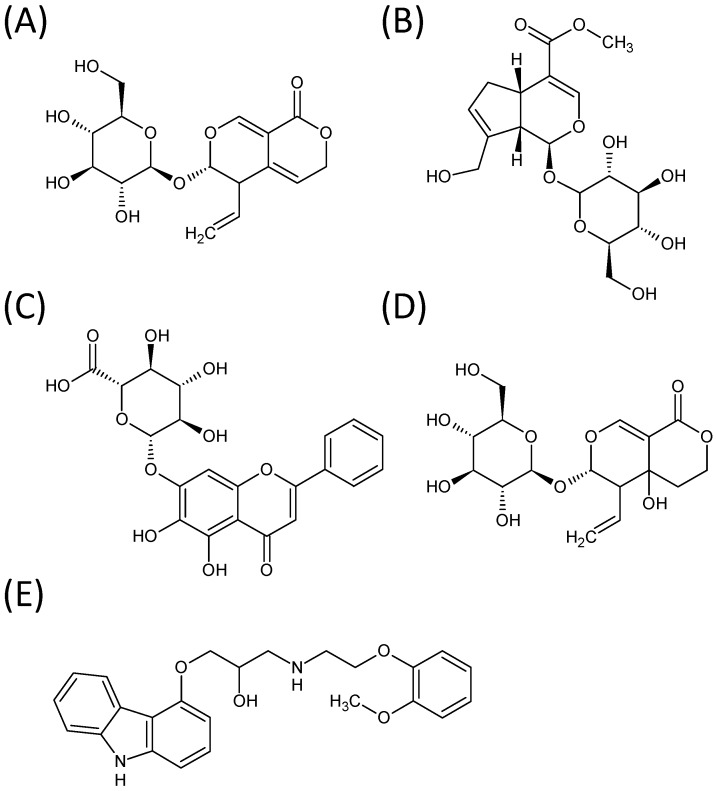
Structure formula of (**A**) gentiopicroside; (**B**) geniposide; (**C**) baicalin; (**D**) swertiamarin; and (**E**) carvedilol (IS).

Although it has been reported that LDXGT has immunomodulatory effects on CD4^+^, CD25^+^ T cells and that it attenuates pathological signs in MRL/lpr mice [3], the bioactive components of the LDXGT compound have not yet been elucidated completely. Some of the components contained in the ingredient herbs, however, have been shown to have pharmacological properties. Several reports have indicated that gentiopicroside, geniposide, baicalin, and swertiamarin (the chemical structures are shown in Figure 1) have an important role in LDXGT. For example, gentiopicroside isolated from *Gentiana* root has been reported to promote the secretion of gastric juices and benefit the stomach [4]. Early pharmacological studies also have shown that gentiopicroside has antibacterial effects and free radical scavenging activity [5]. Furthermore, an animal study has documented that gentiopicroside provides protection against hepatitis induced by chemically and immunologically induced acute hepatic injuries [6]. Geniposide isolated from the fruits of *Gardenia* has shown anti-angiodenic and anti-inflammatory activities [7,8]. Baicalin, the flavonoids isolated from roots of *Scutellaria baicalensis* Georgi, have shown multi-functional efficacies, including antibacterial, antivirus, anti-inflammation and hepatoprotective activities [9,10]. Swertiamarin isolated from the *Gentiana* species has been reported to have important and extensive pharmacological activities, including antibacterial, hepatoprotective, antioxidant, anti-inflammatory, antinociceptive and antispastic properties, according to *in vitro* or *in vivo* pharmacodynamic experiments [11].

Recently, analytical methods have been established for analysis of the bioactive components of LDXGT by two-dimensional liquid chromatography with immobilized liposome chromatography column [12] and high-performance liquid chromatography coupled to photodiode array and electrospray ionization mass spectrometry (HPLC-DAD-ESI-MS) [13]. Although a previous study has investigated the plasma profiles and compared the pharmacokinetics of gentiopicroside in rats after oral administration of gentiopicroside alone, LDXGT, and a signal herb decoction of Radix Gentianae by HPLC [14], there is still limited information on simultaneous determination of the bioactive components of LDXGT by LC-MS/MS as applied to investigate its pharmacokinetics in freely moving rats.

The aim of this study was to develop and validate the LC-MS/MS method for the simultaneous determination of gentiopicroside, geniposide, baicalin, and swertiamarin in rat plasma after oral administration of LDXGT, and to investigate the absorption, distribution, and elimination of the multiple components in a traditional Chinese herbal prescription. It was expected that the four bioactive components of LDXGT would be detected in rat plasma and these herbal components were absorbed in multiple absorption sites or regulated by enterohepatic circulation. Furthermore, the pharmacokinetic study of the above components could help to elucidate the absorption mechanism of LDXGT for additional interpretation of traditional Chinese medicine.

## 2. Results and Discussion

### 2.1. Optimization of LC-MS/MS Conditions

To develop a sensitive and accurate analysis method for the determination of gentiopicroside, geniposide, baicalin, and swertiamarin, a triple quadruple mass spectrometer equipped with an electrospray ionization source is currently one of the most powerful tools for the simultaneous quantification of herbal components because of its high selectivity and sensitivity. Owing to investigation of the full scan and product ion scan mass spectra of analytes, the signal intensity in the positive mode was higher than that in the negative ion mode. Thus, all detection was carried out using the predominantly positive ion mode.

Qualification analysis of a triple-quadrupole mass spectrometer operated in the multiple reaction monitoring (MRM) mode consisting of two parts: selecting the precursor ion (MS 1), and selecting a specific fragment of product ion (MS 2). These two devices generated a very specific and sensitive response for the selected analyte, hence the integrated peak for the target component could be monitored in a sample after a simple one-dimensional chromatographic separation. The main mass fragments of the four active components are listed below: gentiopicroside: *m/z* 357.10 [M+H]^+^→195.10, collision energy: −10.0 eV; geniposide: *m/z* 406.10 [M+NH_4_]^+^→227.10, collision energy: −10.0 eV; baicalin: *m/z* 447.00 [M+H]^+^→271.05, collision energy: −25.0 eV; swertiamarin: *m/z* 375.10 [M+H]^+^→195.10, collision energy: −10.0 eV; and carvedilol (IS): *m/z* 407.20 [M+H]^+^→100.15, collision energy: −35.0 eV, respectively (Table 1). The MS/MS parameters manually obtain the highest response for all of the precursor and product ion combinations.

**Table 1:** The analytical conditions of LC-MS/MS for the identification of the four components.

	Components	Molecular Weight	t_R_ (min)	Mass Fragments		Collision Energy (eV)
				Q1 Mass (amu)	Q3 Mass (amu)	
A	Gentiopicroside	356.32	4.0	357.10 [M+H]^+^	195.10	−10.0
B	Geniposide	388.36	4.1	406.10 [M+NH_4_]^+^	227.10	−10.0
C	Baicalin	446.36	5.9	447.00 [M+H]^+^	271.05	−25.0
D	Swertiamarin	374.12	3.6	375.10 [M+H]^+^	195.10	−10.0
E	Carvedilol (IS)	406.48	5.8	407.20 [M+H]^+ ^	100.15	−35.0

**Figure 2:**
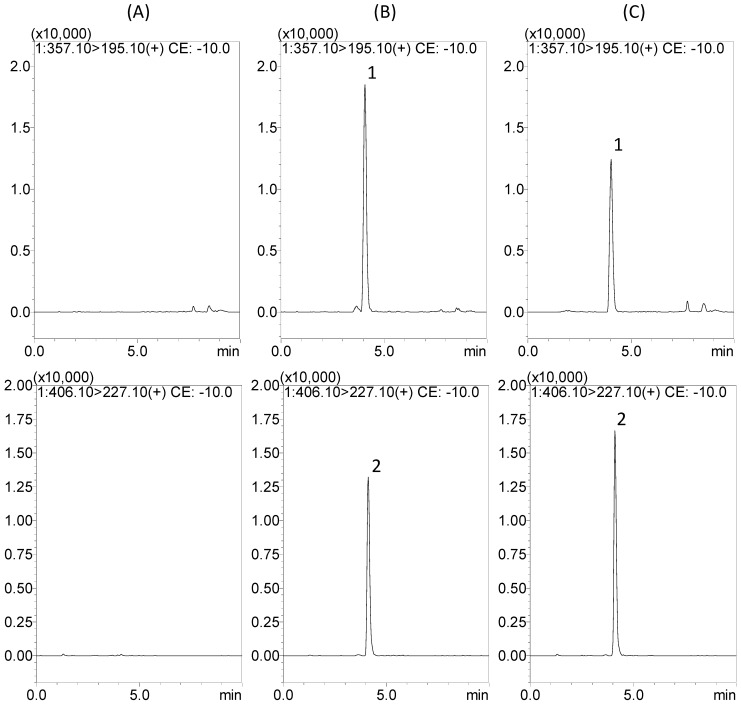

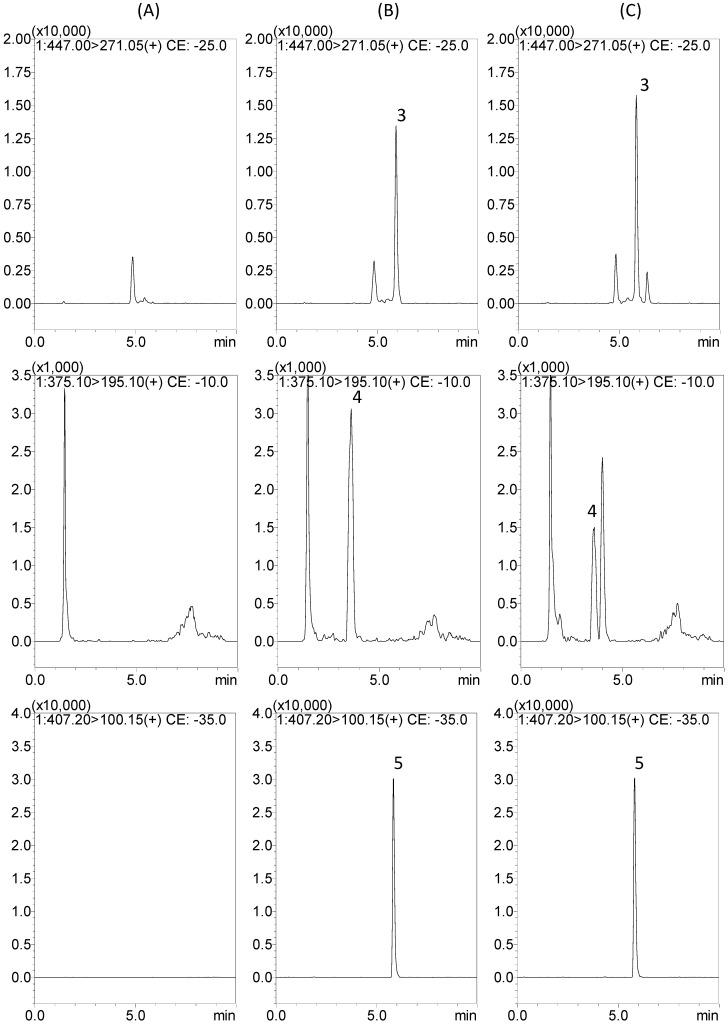
Representative MRM chromatograms of gentiopicroside (channel 1), geniposide (channel 2), baicalin (channel 3), swertiamarin (channel 4), and carvedilol (IS) (channel 5) in rat plasma: (**A**) blank plasma samples; (**B**) blank plasma samples spiked with gentiopicroside, geniposide, baicalin, swertiamarin, and carvedilol (IS) at 500, 50, 100, 25, and 10 ng/mL, respectively; (**C**) diluted plasma sample (×10) of gentiopicroside, geniposide, and baicalin at 240 min, and plasma sample of swertiamarin at 90 min, after oral administration of Long-Dan-Xie-Gan-Tang (10 g/kg, p.o.).

The retention time of gentiopicroside, geniposide, baicalin, swertiamarin, and carvedilol (IS) were 4.0, 4.1, 5.9, 3.6, and 5.8 min, respectively. To determine appropriate retention time, better resolution, and sensitivity, multiple chromatographic conditions were investigated. Several mobile phase systems in different ratios, such as acetonitrile–water and methanol–water, were examined in the course of analytical methods. The gradient elution, proper column, and flow rate were pivotal influences on separation for multi-components. Finally, a gradient system of methanol–water containing 1 mM ammonium acetate with 0.1% formic acid was chosen for the mobile phase in this study. The chromatographic conditions achieved are symmetric peak shape, good resolution, a short runtime (10 min), and appropriate ionization in the presence of endogenous species and co-elution (Figure 2).

### 2.2. Protein Precipitation Methods for Sample Preparation

Since a great number of samples needed to be analyzed for pharmacokinetic analysis, having a simple, rapid, and economic sample preparation method is critical. Protein precipitation was more advisable and advantageous in the present work because it can not only ensure less endogenous interference, adequate recovery, and high sensitivity but also provides simple performance. The estimation of protein precipitation with some modifications was carried out following a previous report [15]. Methanol was more appropriate for reducing the matrix effect than acetonitrile. Satisfactory peak shape, matrix effect, and higher responses were obtained with the 5% formic acid addition. In the course of testing, methanol containing 5% formic acid solution (v/v) was chosen as the precipitation agent.

### 2.3. Method Validation

#### 2.3.1. Selectivity

The selectivity was assessed by comparing the chromatograms of blank plasma samples obtained from six rats with corresponding spiked plasma samples. Figure 2 reveals that no interferences exist in the present analytical conditions. 

#### 2.3.2. Linearity, the Lower Limit of Determination (LLOD) and Lower Limit of Quantification (LLOQ)

For a standard calibration curve, the ratios of the chromatographic peak areas (analytes/internal standard) as ordinate variables were plotted *versus* the concentration of these drugs as abscissa. The linearity of calibration curves were demonstrated by the good determination of coefficients (r^2^) obtained for the regression line. Good linearity was achieved over the calibration range, with all coefficients of correlation greater than 0.995. The mean values of regression equation of the analytes in rat plasma are listed as follows: y = 0.0021x − 0.0013 (r^2^ = 0.9998, gentiopicroside); y = 0.0069x + 0.0172 (r^2^ = 0.9998, geniposide), y = 0.0050x − 0.0019 (r^2^ = 0.9994, baicalin), and y = 0.0086x − 0.0099 (r^2^ = 0.9998, swertiamarin). The linear ranges were 10–500 ng/mL for gentiopicroside, geniposide, and baicalin, and 5–250 ng/mL for swertiamarin in biological samples.

The data showing the LLOD for the four components in rat plasma were gentiopicroside (3 ng/mL), geniposide (1 ng/mL), baicalin (3 ng/mL), and swertiamarin (1 ng/mL). Peak areas in chromatograms for the spiked plasma samples containing the above lowest concentrations were compared with the signal-to-noise ratio ≥ 3. Sensitivity is evaluated by the LLOQ determinations, which are defined as the lowest concentration that can be reliably and reproducibly measured in at least three replicates. The LLOQ values for the four components in rat plasma were gentiopicroside (10 ng/mL), geniposide (5 ng/mL), baicalin (10 ng/mL), and swertiamarin (5 ng/mL). Peak areas in the chromatograms for the spiked plasma samples containing the above lowest concentrations were compared with the signal-to noise ratio ≥ 10.

#### 2.3.3. Precision and Accuracy

The intra- and inter-day precision and accuracy were determined by measuring six replicates of QC samples at six concentration levels. The performance data of the assay is summarized in Table 2. The intra- and inter-day precision (RSD, %) of four bioactive components ranged from 0.9% to 11.4% and from 0.3% to 14.4%, respectively. The accuracy (relative error, RE) was from −6.3% to 10.1% at all QC levels. These results are within the acceptable criteria for the FDA Bioanalytical Method Validation guidelines and show that the LC-MS/MS method was accurate, reliable, and reproducible for the quantitative analysis of gentiopicroside, geniposide, baicalin, and swertiamarin in rat plasma.

**Table 2:** Intra-day and inter-day precision and accuracy for the determination of four components.

Nominal conc.(ng/mL)	Intra-day			Inter-day		
	Observed conc. (ng/mL)	Precision, RSD (%)	Accuracy, Bias (%)	Observed conc. (ng/mL)	Precision, RSD (%)	Accuracy, Bias (%)
Gentiopicroside						
10	9.82 ± 0.98	10.0	−1.8	10.1 ± 1.45	14.4	0.9
25	23.5 ± 2.68	11.4	−6.0	25.7 ± 2.11	8.2	2.9
50	49.9 ± 4.29	8.6	−0.3	49.2 ± 1.26	2.6	−1.6
100	98.1 ± 2.52	2.6	−1.9	99.6 ± 2.91	2.9	−0.4
250	259 ± 6.67	2.6	3.5	251 ± 5.72	2.3	0.3
500	494 ± 5.09	1.0	−1.1	500 ± 2.82	0.6	−0.1
Geniposide						
10	9.37 ± 0.88	9.4	−6.3	9.77 ± 1.16	11.9	−2.3
25	25.4 ± 1.19	4.7	1.5	26.0 ± 1.96	7.5	3.9
50	49.5 ± 1.01	2.0	−0.9	49.8 ± 3.06	6.1	−0.4
100	98.2 ± 4.04	4.1	−1.8	100 ± 3.02	3.0	0.1
250	256 ± 5.66	2.2	2.3	249 ± 3.50	1.4	−0.4
500	497 ± 7.04	1.4	−0.5	500 ± 1.48	0.3	0.1
Baicalin						
10	11.0 ± 1.01	9.1	10.1	10.2 ± 1.51	14.8	2.1
25	24.9 ± 1.65	6.6	−0.3	25.8 ± 2.70	10.5	3.3
50	50.9 ± 1.92	3.8	1.9	47.3 ± 1.12	2.4	−5.4
100	98.4 ± 4.51	4.6	−1.6	99.3 ± 3.04	3.1	−0.7
250	245 ± 8.61	3.5	−1.9	250 ± 4.71	1.9	−0.1
500	491 ± 19.6	4.0	−1.7	505 ± 9.20	1.8	1.0
Swertiamarin						
5	5.35 ± 0.33	6.2	7.0	4.74 ± 0.25	5.3	−5.1
10	9.91 ± 0.66	6.7	−0.9	10.1 ± 0.53	5.2	1.0
25	24.8 ± 0.37	1.5	−0.9	24.9 ± 0.75	3.0	−0.4
50	50.0 ± 1.06	2.1	−0.1	49.8 ± 1.16	2.3	−0.4
100	99.6 ± 1.28	1.3	−0.4	101 ± 1.35	1.3	0.9
250	251 ± 2.20	0.9	0.5	253 ± 7.21	2.9	1.1

Data are expressed as mean ± S.D. (*n* = 6).

#### 2.3.4. Matrix Effect and Recovery

To measure the matrix effect, it was determined at three different concentrations for all analytes. An absolute matrix effect was observed for some of the analytes at some of the low, middle, and high concentrations. Nevertheless, no relative matrix effects were seen, since the coefficients of variation at each concentration level were within ±20% for all components except geniposide (116.9% ± 6.9%) and baicalin (85.0% ± 13.6%), which were slightly out of range at 10 ng/mL. The ion suppression/enhancement in signal ranged substantially between 80% and 120% for the three different levels, indicating that the matrix effect on the ionization of analytes is not obvious under those conditions.

The extraction recoveries were also determined for three replicates from rat plasma spiked with low, middle, and high concentrations of the four components and internal standard. The mean recoveries of most samples were within 80%–120%, except geniposide. The recovery of geniposide was 119.4% ± 7.9% at a low concentration of 10 ng/mL. Even though it was slightly out of range, the extraction recovery of geniposide was stable and acceptable. The matrix effect and extraction recoveries from rat plasma are shown in Table 3.

**Table 3:** Matrix effect and extraction recovery of the four components and internal standard in rat plasma after sample preparation.

Nominal conc.		Peak Area		Matrix Effect	Recovery
(ng/mL)	Set 1	Set 2	Set 3	(%) ^a^	(%) ^b^
Gentiopicroside					
10	3911 ± 321	4001 ± 595	4312 ± 285	102.0 ± 7.4	108.7 ± 9.2
50	17829 ± 774	20372 ± 283	21498 ± 1749	114.4 ± 3.6	105.6 ± 9.9
250	89698 ± 3141	93932 ± 2505	104970 ± 4074	104.8 ± 4.1	111.7 ± 2.5
Mean ± S.D.				107.0 ± 7.3	108.7 ± 7.3
Geniposide					
10	12233 ± 190	14306 ± 1062	17028 ± 644	116.9 ± 6.9	119.4 ± 7.9
50	58115 ± 1045	67062 ± 1062	71407 ± 1764	115.4 ± 1.6	106.5 ± 1.1
250	295991 ± 3955	316422 ± 6847	346662 ± 8807	106.9 ± 3.7	109.6 ± 0.6
Mean ± S.D.				113.1 ± 6.1	111.8 ± 7.1
Baicalin					
10	9173 ± 232	7795 ± 1246	7825 ± 1054	85.0 ± 13.6	101.1 ± 10.7
50	45932 ± 1992	39518 ± 2684	37717 ± 460	86.0 ± 3.1	95.7 ± 5.6
250	224485 ± 4019	198185 ± 9541	186908 ± 6196	88.3 ± 3.2	94.4 ± 2.8
Mean ± S.D.				86.4 ± 7.3	97.1 ± 6.9
Swertiamarin					
10	16564 ± 490	16634 ± 397	17422 ± 660	100.5 ± 4.5	104.7 ± 1.7
50	76343 ± 1289	82585 ± 5095	88245 ± 4264	108.1 ± 4.8	107.0 ± 5.2
250	404293 ± 4777	432683 ± 3140	467239 ± 7470	107.0 ± 0.5	108.0 ± 1.0
Mean ± S.D.				105.2 ± 4.9	106.6 ± 3.1
Carvedilol (IS)					
10	76558 ± 6622	87040 ± 5353	83146 ± 3942	113.9 ± 2.9	95.7 ± 4.7

Data are expressed as mean ± S.D. (*n* = 3); ^a^ Matrix effect expressed as the ratio of the mean peak area of an analyte spiked post extraction (set 2) to the mean peak area of the same analyte standard (set 1) multiplied by 100. A value of >100% indicates ionization enhancement, and a value of <100% indicates ionization suppression; ^b^ Recovery calculated as the ratio of the mean peak area of an analyte spiked before extraction (set 3) to the mean peak area of an analyte spiked post extraction (set 2) multiplied by 100.

#### 2.3.5. Stability

The stability of gentiopicroside, geniposide, baicalin, and swertiamarin in rat plasma was evaluated by using low, medium, and high concentrations of analytes. The four components were generally stable under the storage and analytical process conditions, including three freeze-thaw cycles, short-term (room temperature for 3 h), long-term (−20 °C for 7 days) and autosampler stability (8 °C for 12 h) in biological samples, and the deviation of the mean measured three different concentrations (10, 50, and 250 ng/mL) of the samples from the nominal concentration within ±15% of the initial values (Table 4). These stability results show no significant differences between the initial concentration and these QC samples.

**Table 4:** Stability of gentiopicroside, geniposide, baicalin, and swertiamarin in rat plasma QC samples.

Analytes/	Freeze-thaw Stability		Short-term Stability		Long-term Stability		Autosampler Stability	
Spiked Concentration (ng/mL)	Measured Concentration (ng/mL)	Accuracy (%)	Measured Concentration (ng/mL)	Accuracy (%)	Measured Concentration (ng/mL)	Accuracy (%)	Measured Concentration (ng/mL)	Accuracy (%)
Gentiopicroside								
10	11.3 ± 0.28	113.0	11.0 ± 0.40	110.2	9.82 ± 0.21	98.2	11.0 ± 1.09	109.8
50	53.7 ± 1.44	107.4	55.5 ± 1.68	111.0	51.1 ± 2.71	102.2	49.0 ± 2.47	97.9
250	265 ± 6.60	106.0	263 ± 16.2	105.1	240 ± 6.72	95.9	253 ± 10.7	101.1
Geniposide								
10	10.0 ± 0.94	100.3	11.4 ± 0.80	114.1	9.10 ± 0.77	91.1	10.3 ± 0.65	102.8
50	56.5 ± 1.00	113.0	52.9 ± 1.12	105.8	46.4 ± 4.16	92.7	48.9 ± 1.86	97.7
250	265 ± 10.9	106.0	260 ± 6.85	103.9	235 ± 15.4	94.1	248 ± 5.47	99.4
Baicalin								
10	10.3 ± 1.01	103.0	11.2 ± 1.14	111.8	9.54 ± 0.97	95.4	9.28 ± 0.72	92.8
50	49.5 ± 0.75	99.0	48.4 ± 1.18	96.7	49.7 ± 6.84	99.5	50.5 ± 4.33	101.0
250	231 ± 12.0	92.2	231 ± 10.8	92.6	238 ± 33.2	95.3	231 ± 1.57	92.4
Swertiamarin								
10	10.6 ± 0.76	105.7	11.3 ± 0.99	112.7	9.50 ± 0.50	95.0	10.6 ± 0.78	106.1
50	49.8 ± 0.66	99.6	48.5 ± 0.23	96.9	45.7 ± 3.21	91.3	49.9 ± 1.08	99.7
250	247 ± 2.74	98.7	252 ± 8.03	100.9	224 ± 10.7	89.8	240 ± 3.52	96.1

Data are expressed as mean ± S.D. (*n* = 3). The accuracy (%) was calculated as follows: Accuracy (%) = C_obs_/C_nom_ × 100%; Freeze-thaw stability: three freeze-thaw cycles; Short-term stability: room temperature for 3 h; Long-term stability: kept at −20 °C for 7 days; Autosampler stability: 8 °C for 12 h at the autosampler.

### 2.4. Application of the Analytical System in Pharmacokinetic Study

**Figure 3:**
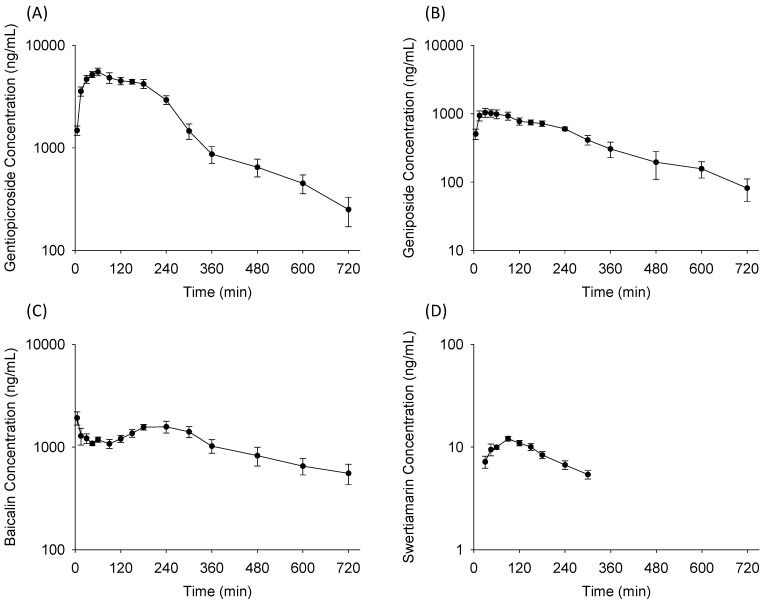
Mean plasma concentration-time profile of (**A**) gentiopicroside; (**B**) geniposide; (**C**) baicalin; and (**D**) swertiamarin in rat plasma after oral administration of Long-Dan-Xie-Gan-Tang (LDXGT; 10 g/kg). The herbal contents of LDXGT are gentiopicroside 17.04 mg/g, geniposide 25.08 mg/g, baicalin 9.94 mg/g, and swertiamarin 0.22 mg/g. Data are expressed as mean ± S.E.M. (*n* = 6).

The validated LC-MS/MS method was successfully applied for the pharmacokinetic study of the four active components after LDXGT administration (10 g/kg, p.o.) in freely moving rats. The mean plasma concentration-time profiles are illustrated in Figure 3 and the pharmacokinetic parameters are presented in Table 5.

Gentiopicroside showed a single absorption phase in the concentration-time curves and was rapidly absorbed with detectable levels of gentiopicroside at 5 min in rat plasma after LDXGT oral administration [15]. A previous study also indicated that gentiopicroside could be absorbed immediately in mice [16]. After rapidly achieving maximal levels of plasma concentrations, gentiopicroside declined sharply and was followed by a slower phase of decrease until 4 h. Moreover, the pharmacokinetic parameters of gentiopicroside showed the largest C_max_ at 5767 ± 412 ng/mL (*p* < 0.05) and AUC at 1417 ± 83.8 min µg/mL (*p* < 0.05) among the four bioactive components after LDXGT administration.

**Table 5:** Pharmacokinetic parameters of the four components in rat plasma after oral administration of LDXGT (10 g/kg, p.o.).

Parameter	Gentiopicroside	Geniposide	Baicalin	Swertiamarin
C_max_ (ng/mL)	5767 ± 412 ^a^	1164 ± 144	2008 ± 265	13.0 ± 0.5
T_max_ (min)	60.0 ± 6.7	55.0 ± 12.6	15.8 ± 9.0	97.5 ± 14.4
t_1/2_ (min)	127 ± 7.7	168 ± 27.4	314 ± 56.3	188 ± 27.6
AUC (min µg/mL)	1417 ± 83.8 ^b^	302 ± 25.8	753 ± 86.2	2.5 ± 0.1
Cl (mL/min/kg)	118 ± 6.5	784 ± 55.5	112 ± 20.7	581 ± 58.5
MRT (min)	193 ± 7.9	213 ± 18.2	298 ± 11.6	144 ± 3.1

Data expressed as mean ± S.E.M. (*n* = 6); C_max_: the maximum plasma concentration, T_max_: time to reach the maximum concentrations, t_1/2_: half-life, AUC: area under the concentration-time curve, CL: clearance, MRT: mean resident time; ^a^ Significantly different (*p* < 0.05) from the other active components in C_max_; ^b^ Significantly different (*p* < 0.05) from the other active components in AUC.

Geniposide displayed a plateau absorption phase in the concentration-time curves and showed a slower phase of elimination in our pharmacokinetic profiles. Although the content of geniposide (25.08 mg/g) was higher than gentiopicroside (17.04 mg/g) in LDXGT, geniposide did not show the largest C_max_ (1164 ± 144 ng/mL) and AUC (302 ± 25.8 min µg/mL) in pharmacokinetics. There are many possible explanations, and one of the influencing factors might be that the relative bioavailability of gentiopicroside (10.3%) was higher than geniposide (4.2%) [15,17]. In addition, the previous results of Sun *et al*. [18] illustrated that the oral bioavailability of geniposide was dramatically enhanced in different combinations of its constituent herbs. It can be deduced that the absorption and oral bioavailability of geniposide in rats may significantly vary from herb–herb interaction [17,19].

Swertiamarin showed the lowest level of AUC (2.5 ± 0.1 min µg/mL) and fell below the lower limit of quantification (LLOQ) at 300 min in the concentration-time curves after LDXGT administration. The data of Li *et al*. [20] also demonstrated that swertiamarin showed rapid absorption and elimination, and high concentrations were found in the liver and kidneys, indicating that swertiamarin was rapidly metabolized in the liver and eliminated by the kidneys. Furthermore, Wang *et al*. [21] previously reported that swertiamarin was biotransformed to erythrocentaurin and 3,4-dihydro-5-(hydroxymethyl) isochroman-1-one by human intestinal bacteria. It was assumed that this low level in rat plasma was due to either the low content in LDXGT (0.22 mg/g) or the first pass effect. The possible reasons should be confirmed in our further work and we will seek to identify and detect the major metabolites of swertiamarin *in vivo*.

Baicalin displayed rapid and sustained absorption (T_max_ = 15.8 ± 9.0 min, t_1/2_ = 314 ± 56.3 min). The concentration-time curves of baicalin presented a double-peak absorption phase in the plasma profile. There were many possible explanations for the phenomenon of multiple-peak behavior. Shaw *et al*. [22] indicated that several mechanisms have been proposed for the phenomenon: (1) enterohepatic recycling; (2) the presence of absorption sites along the stomach and different gastrointestinal segments; and (3) variable gastric emptying. Furthermore, the pharmacokinetic results of Zhang *et al*. [23] also demonstrated that baicalin presented a bimodal phenomenon in the plasma profile, and it is generally assumed that baicalin is poorly absorbed from the gastrointestinal tract in its native form and must be hydrolyzed by microflora enzymes (bacterial glucuronidase) in the gut into aglycone-baicalein, and then reconverted back to baicalin. Our pharmacokinetic results indicate that there would be multiple absorption sites or regulation of enterohepatic circulation of baicalin in rats.

In this study, we first focused our investigation on the pharmacokinetic profile of major bioactive components in *Gentiana scabra* (gentiopicroside and swertiamarin), *Gardenia jasminoides* (geniposide), and *Scutellaria baicalensis* (baicalin). The validated LC-MS/MS method was successfully applied to a pharmacokinetic study in freely moving rats after administration of a Chinese herbal prescription of Long-Dan-Xie-Gan-Tang.

## 3. Experimental

### 3.1. Chemicals and Reagents

The chemicals gentiopicroside and swertiamarin were purchased from Nacalai Tesque, Inc. (Kyoto, Japan). Geniposide, baicalin, carvedilol (internal standard, IS), pentobarbital, and heparin were obtained from Sigma-Aldrich Chemicals (St. Louis, MO, USA). Ammonium acetate, formic acid, methanol of HPLC grade, and other reagents were purchased from E. Merck (Darmstadt, Germany). Triply deionized water was prepared by Millipore (Milford, MA, USA) and used for all preparations in this study.

### 3.2. Herbal Preparation of Long-Dan-Xie-Gan-Tang

The 10 herbs of LDXGT were purchased from a Chinese traditional herbal medicine store in Taipei and prepared in the National Research Institute of Chinese Medicine, Taipei, Taiwan. According to a previous study on the preparation of herbal formulas [22], the crushed herbs of *Gentiana scabra* (69.3 g), *Scutellaria baicalensis* (34.6 g), *Gardenia jasminoides* (34.6 g), *Alisma orientalis* (69.3 g), *Clematis montana* (34.6 g), *Plantago asiatica* (34.6 g), *Angelica sinensis* (34.6 g), *Rhemannia glutinosa* (34.6 g), *Bupleurum chinense* (69.3 g), and *Glycyrrhiza uralensis* (34.6 g) were co-boiled with 9000 mL water in a water bath at 70 °C for 9 h and filtered. This process was repeated once and then the filtrates were combined and concentrated to 500 mL by rotary evaporator at 60 °C. The extracted solution was evaporated under vacuum and partitioned. Water was removed by freeze-drying. The above crushed herbs were extracted to 144.65 g, so the extraction yield of the decoction was about 32.14% (144.65:450.00, w/w). The lyophilized powder of LDXGT was used for the following experiment.

To calculate the administration dosage, the contents of gentiopicroside, geniposide, baicalin, and swertiamarin in LDXGT extract were determined by LC-MS/MS with the same analytical conditions used in Section 3.3. The results demonstrated that the contents of the four components of LDXGT were quantitated as follows (mg/g): gentiopicroside 17.04 mg/g, geniposide 25.08 mg/g, baicalin 9.94 mg/g, and swertiamarin 0.22 mg/g in LDXGT.

### 3.3. LC-MS/MS and Analytical Conditions

The LC-MS/MS analysis was performed using a Shimadzu LCMS-8030 triple-quadrupole mass spectrometer equipped with an electrospray ionization (ESI) interface and coupled to the UFLC system, equipped with two chromatographic pumps (LC-20AD XR), an autosampler (SIL-20AC XR), a DGU-20A5 degasser, a forced air-circulation-type column oven (CTO-20A), and a photo-diode array detector (SPD-M20A) (Shimadzu, Kyoto, Japan). The optimized instrument settings were as follows: interface voltage, 4.5 kV; desolvation line temperature, 250 °C; heat block temperature, 400 °C; desolvation gas, nitrogen; desolvation gas flow rate, 3 L/min; drying gas, nitrogen; drying gas flow rate, 17 L/min; collision gas, argon; and collision gas pressure, 230 kPa.

The MS/MS measurements operating parameters were performed in the multiple reaction monitoring (MRM) mode. The chromatographic separation was achieved using a Acquity UPLC BEH C18 column (2.1 mm × 100 mm, 1.7 μm, Waters Corporation) with a KrudKatcher Ultra In-Line Filter (AF0-8497, 0.5 µm Depth Filter × 0.004 in, Phenomenex Corporation), and the column temperature was maintained at 35 °C. The mobile phase consisted of A (1 mM ammonium acetate and 0.1% formic acid in water) and B (0.1% formic acid in methanol) with a linear gradient elution of 20%–95% (v/v) B at 0–5 min, 95% B at 5–7 min, and 20% B at 7–10 min. The flow rate was 0.2 mL/min and the injection volume was 5 μL.

### 3.4. Preparation of Calibration Standards and Quality Controls

The stock solutions were prepared by dissolving 1.0 mg each of gentiopicroside, geniposide, baicalin, and swertiamarin into 1.0 mL of 100% methanol to a final concentration of 1.0 mg/mL. All stock solutions were stored at −20 °C before use. A series of working standard solutions was freshly prepared by spiking aliquots of the stock solutions into drug-free plasma samples to obtain the following concentrations: 1, 5, 10, 25, 50, 100, 250, 500, and 1000 ng/mL. Working solutions for QC samples with low, medium, and high concentrations were prepared in the same manner.

### 3.5. Method Validation

Full validation of the analytical method estimated in this study was according to the US Food and Drug Administration guidelines for validation of bioanalytical methods [24]. The analytical method was considered valid according to assays of its selectivity, linearity, accuracy, precision, lower limit of quantification (LLOQ), matrix effect, extraction recovery, and stability.

#### 3.5.1. Selectivity

The selectivity was assessed by comparing the chromatograms of blank plasma samples obtained from six rats with corresponding spiked plasma samples.

#### 3.5.2. Linearity, the Lower Limits of Detection (LLOD) and Lower Limits of Quantification (LLOQ)

The sample preparation for calibration curves involved creating freshly spiked plasma (45 μL) samples with stock working solution (5 μL) of analytes at concentration ranges of 5–1000 ng/mL and extracted as described in Section 3.6.4. The concentration of each sample was derived from the calibration curve and corrected by the respective dilution volume. For a standard curve, the ratios of the chromatographic peaks area (analytes /internal standard) as ordinate variables were plotted *versus* the concentration of these drugs as abscissa. All linear curves were required to have a coefficient of estimation of at least > 0.995. The lower limits of detection (LLOD) and lower limits of quantification (LLOQ) were determined at a signal-to-noise ratio of about 3 and 10 by analyzing the diluted standard solution.

#### 3.5.3. Precision and Accuracy

The intra- and inter-day variability for the four analytes were determined by six replicates at concentrations of 5, 10, 25, 50, 100, 250, 500, and 1000 ng/mL using the LC-MS/MS method described above on the same day (intra-day) and six different days (inter-day), respectively. The accuracy (bias, %) was calculated from the mean value of observed concentration (C_obs_) and nominal concentration (C_nom_) using the relationship accuracy (Bias, %) = [(C_obs_ − C_nom_)/C_nom_] × 100%. The relative standard deviation (RSD, %) was calculated from the observed concentrations as precision (RSD, %) = [standard deviation (SD)/C_obs_] × 100%. Accuracy and precision values within ±15% were considered acceptable in the experimental concentration range, and the LLOQ values were all less than ±20%.

#### 3.5.4. Matrix Effect and Recovery

Following the previous report of Hou *et al*. [25], three sets of extraction methods were prepared to evaluate the matrix effect and recovery in the quantitative bioanalytical method.

*Set 1.* Standard solutions were constructed using neat standard solutions in the mobile phase. The samples were prepared by placing 5 μL of the appropriate concentrations of standard solutions and 145 μL of the mobile phase (150 μL, total volume) into 1.5-mL centrifuge tubes. After mixing, the solutions were transferred to autosampler vials, and 5 μL was injected directly into LC-MS/MS system.

*Set 2.* Standard solutions spiked after extraction were constructed in three different lots of blank plasma by placing 50 μL of plasma in 1.5-mL centrifuge tubes, followed by the addition of 100 μL methanol (containing 5% formic acid, v/v) for protein precipitation (vortex 5 min). After centrifugation (13,100× *g* for 10 min, at 4 °C), the supernatant was filtered using a 0.22-μm mini syringe filter. The supernatant (145 μL) was supplemented with 5 μL of appropriate concentrations of standard solutions. After mixing, the solutions were transferred to autosampler vials, and 5 μL was injected into LC-MS/MS for analysis. In *set 2*, the standard solutions were spiked after extraction into different lots of plasma, whereas in *set 3*, the standard solutions were spiked into different lots of plasma before extraction.

*Set 3.* The standard solutions spiked before extraction were constructed in three different lots of plasma by placing 45 μL of plasma in 1.5-mL centrifuge tubes to which 5 μL of appropriate concentrations of standard solutions were added before extraction, followed by the addition of 100 μL of methanol (containing 5% formic acid, v/v) for protein precipitation (vortex 5 min). After centrifugation (13,100× *g* for 10 min, at 4 °C), the supernatant was filtered using a 0.22-μm mini syringe filter. Finally, the solutions were transferred to autosampler vials, and 5 μL was injected into LC-MS/MS for analysis.

By comparing the peak areas of the standard solutions, standard solutions spiked before and after extraction into different lots of plasma at low, medium, and high concentrations using three replicates, the recovery and ion suppression or enhancement associated with a given lot of plasma were assessed.

Results obtained in this manner were used to determine the matrix effect (ME) and recovery (RE) of the extraction. The peak areas obtained in neat standard solutions in *set 1* were indicated as A, the corresponding peak areas for standard spiked after extraction into plasma or feces homogenate extracts as B (*set 2*), and the areas for standard spiked before extraction as C (*set 3*); the ME and RE values could be calculated as follows: ME (matrix effect, %) = (B/A) × 100%; RE (recovery, %) = (C/B) × 100%.

#### 3.5.5. Stability

To estimate the stability of analytes in rat plasma during the experiments, storage, and analysis processes, spiked samples with low, median, and high concentrations of analytes were designed and conducted under different conditions, including freeze-thaw cycle analysis, short-term stability, long-term stability, and autosampler stability. Freeze-thaw stability was assessed over three freeze and thaw cycles. Short-term stability was determined by keeping the samples at room temperature for 3 h. Long-term stability was evaluated by analyzing samples kept at −20 °C for 7 days. Autosampler stability was determined for samples kept at the autosampler temperature (8 °C) for 12 h. All stabilities were calculated as the ratio of average concentration and freshly prepared samples (*n* = 3). The stability (accuracy, %) was calculated as follows: stability (%) = (C_obs_/C_nom_) × 100%.

### 3.6. Pharmacokinetic Application

#### 3.6.1. Experimental Animals

All experimental protocols involving animals were reviewed and approved by the Institutional Animal Care and Use Committee (IACUC) of National Yang-Ming University, Taipei, Taiwan. (IACUC Approval No: 1021003). Male Sprague Dawley rats (200–260 g, 6–7 weeks of age) were obtained from the Laboratory Animal Center at National Yang-Ming University, Taipei, Taiwan. The animals were specifically pathogen-free and housed in standard cages kept in a temperature-controlled room with a 12-h light/dark cycle, and free access to food (Laboratory Rodent Diet 5001, PMI Feeds, Richmond, IN, USA) and water *ad libitum*.

#### 3.6.2. Freely Moving Rat Model

A freely moving rat model was used in this experiment to avoid the stress caused by restraint or anesthesia. Sprague Dawley rats were initially anesthetized with pentobarbital (50 mg/kg, i.p.), and polyethylene tubing (PE-50) was implanted in the right carotid artery for blood sampling. The PE-50 tube was exteriorized, fixed in the dorsal region of the neck. Patency of the tube was maintained by flushing with heparinized saline (20 IU/mL). During the period of surgery, the body temperature of the rats was maintained at 37 °C with a heating pad. After surgery, the rats were kept in cages individually and allowed to recover for one day.

#### 3.6.3. LDXGT Administration and Sample Collection

The dose of LDXGT (10 g/kg, p.o.) was appropriate for oral administration in rats. About 200 μL of blood samples were withdrawn from the cannula implanted in the carotid artery and placed into a heparin-rinsed vial at 0, 5, 15, 30, 45, 60, 90, 120, 150, 180, 240, 300, 360, 480, 600, and 720 min in 6 healthy rats after a single administration of LDXGT (10 g/kg, p.o.). Then the samples were centrifuged at 3000× *g* for 10 min at 4 °C and the separated plasma samples were frozen in polypropylene tubes at −20 °C until analysis.

#### 3.6.4. Sample Preparation

Plasma samples were prepared with protein precipitation. An aliquot of 50 μL plasma sample and 100 μL methanol (containing 5% formic acid solution and 10 ng/mL of internal standard, v/v) were combined and vortexed for 5 min. The mixture was centrifuged at 13,100× *g* for 10 min at 4 °C. The supernatants were collected, filtered using a 0.22-µm mini syringe filter, and transferred to autosampler vials. Finally, 5 μL was injected into the LC-MS/MS system. If the concentration of analyte was not within the linearity range, the plasma sample was diluted to the appropriate concentration with drug-free plasma and the concentration of analyte was obtained using back-calculation.

### 3.7. Statistical Analysis

Pharmacokinetic calculations were performed on each individual set of data using the pharmacokinetic software WinNonlin Standard Edition, version 1.1 (Scientific Consulting Inc., Apex, NC, USA). A non-compartmental analysis was applied to obtain blood pharmacokinetic parameters, including maximum plasma concentration (C_max_), time to reach the maximum concentrations (T_max_), half-life (t_1/2_), area under concentration-time curve (AUC), and clearance (CL). Statistics and graphics were performed with version 10.0 (SPSS, Chicago, IL, USA) and SigmaPlot 8.0 software. Data were expressed as the mean ± S.D. or mean ± S.E.M. Comparisons were performed using one-way analysis of variance (ANOVA) followed by Dunnett’s test, and the difference was considered statistically significant if *p* < 0.05.

## 4. Conclusions

In this study, a rapid, sensitive, and selective LC-ESI-MS/MS method has been developed and validated for the quantitation of gentiopicroside, geniposide, baicalin, and swertiamarin in rat plasma after oral administration of LDXGT. These pharmacokinetic studies were performed through an experimental model of freely moving rats. The assay provided adequate matrix effect and recovery with good precision and accuracy. These pharmacokinetic results reveal a constructive contribution to comprehending the multiple components of the extensive action mechanism of absorption, distribution, and excretion of a traditional Chinese herbal prescription. Furthermore, the pharmacokinetic profile provides a firm basis for the design of dosing regimens that can contribute to preclinical studies of herbal applications and pharmacological experiments.
